# Bipolar Disorder: an impossible diagnosis

**DOI:** 10.1186/1745-0179-5-13

**Published:** 2009-06-16

**Authors:** Carlo Faravelli, Silvia Gorini Amedei, Maria Alessandra Scarpato, Luca Faravelli

**Affiliations:** 1 Department of Psychology, University of Florence, Firenze, Italy; 2 Department of Neurology and Psychiatry, University of Florence, Firenze, Italy; 3 Department of Psychiatry, University of Pisa, Pisa, Italy

## Abstract

Following the recent debates on the discrepancy between the predominant weight of bipolar disorder (BPD) in the clinical reality and its relatively low prevalence figures emerging from epidemiological surveys, the present paper contends the ability of current operational diagnostic system to properly detect the clinical entity of bipolar disorder.

As an episode of mania/hypomania is the necessary requirement for a diagnosis of bipolar disorder to be made, in this editorial we maintain that: a) the most severe forms of mania, characterized by cloudy consciousness, mood incongruent delusions, and physical symptoms are likely to escape DSM IV criteria, that are shaped around hypomania or mild mania; b) the impossibility to diagnose mania when this occurs during antidepressant treatments impedes diagnosing those cases whose natural illness pattern is Depression followed by Mania (known as DMI pattern); c) given that approximately 50% of cases have their onset of BPD with affective episodes other than mania/hypomania any prevalence figure necessarily underestimates BPD; d) the sub-threshold forms of BPD, well described in the concept of Bipolar Spectrum, are beyond the possibility to be recognized using operational diagnoses in spite of their utmost clinical relevance.

## Commentary

Two commentaries have recently appeared on this journal pointing out the discrepancy between the predominant weight of bipolar disorder in the clinical reality and its relatively low prevalence figures as emerging from the epidemiological surveys [1,2]. The ability of lay interviewers to properly detect patients suffering from Bipolar Disorder (BPD) has been questioned. Moreover, the authors conclude that the current diagnostic thresholds for hypomania and mixed states are unsatisfactory.

In this editorial we will contend that DSM IV, as well as other operational diagnostic systems, is unable to diagnose the clinical entity of BPD.

This issue is based on four points.

### Mania

It is clear that the symptoms chosen by DSM IV as descriptors of a manic state (*Self esteem or grandiosity, Decreased need for sleep, More talkative than usual, Flight of ideas or pressure to keep talking, Distractibility, Increase in activities, Excessive involvement in activities with potentially painful consequences)*[3] are shaped around a prototype of cheerfulness, joy, hyperactivity, grandiosity, elation, inconsideration of the consequences that considers mania as opposite to depression. This contrasts with the classical descriptions of mania as they come from the original accounts of the leading psychopathologists. In a textbook which constituted the basic reading of a couple of generations of psychiatrists, severe mania is described as follows: *"Manic excitement in its most severe form leads to confusion, in which the typical symptoms of mania are obscured. Consciousness, which is clear in the less severe states, becomes clouded, illusions and hallucinations may be observed, and the condition may resemble a delirium. These states are seriously debilitating and may endanger life." (*Mayer-Gross, Slater & Roth, 1960)[4]. Such a presentation would not meet the DSM IV criteria for manic episode. Even other typical descriptions of the manic episode, as those reporting akinetic mania, manic stupor, the presence of symptoms later referred to as first rank symptoms, pseudoparanoid presentations of mania, all described by Kraepelin [5] as varieties of the ever changing paradigm of mania, would be difficult to be included in the DSM IV category of Manic Episode. Even more astonishing, what has represented for years the model of the natural course of the manic episode, brilliantly described by Carlsson and Goodwin in the early 70s [6], with their classical three stages of Mania, would now encounter difficulties in being diagnosed by DSM IV criteria. In fact only stage I, and partially stage II, would fully meet the criteria for mania. The following is another classical description of severe mania that would probably escape DSM IV requirements: "*In the more acute manic reactions the patient, driven by a greater pressure of activity, terror and excitement, becomes violent, attacks his neighbours or begins to shout all kinds of accusations against his alleged persecutors..... Distortions, misinterpretations and ideas of reference are now elaborated into delusions of persecution accompanied by violence and panic. The patient runs down the street nude, sets fire to the house, starts an argument with the police, .......If crossed or interfered with in any way he becomes abusive, destructive*.." (Campbell, 1953)[7].

Even with the understanding that operational diagnostic criteria are aimed at diagnosing mania and not at describing the entire variety of its clinical presentations, it seems that DSM IV misses some of the most typical features of mania (e.g. lability of affect, rapid variations of mood, coexistence of different emotional states). It is only able, therefore, to detect and classify a limited number of the clinical presentations of Mania, and possibly not the actually typical ones. In particular, it seems that diagnostic criteria fail to consider the most severe forms of mania, where the initial features of cheerfulness and grandiosity give way to psychotic ideation, dysphoric affect, mood incongruent delusions and clouding of conscience.

The problem would be even greater with mixed states.

### Antidepressant Induced mania

DSM IV states that the manic presentation secondary to drug treatment cannot be diagnosed as Manic episode (*criterion E): The symptoms are not due to the direct physiological effects of a substance (e.g., a drug of abuse, a medication or other treatment*)[3].

On one hand, a largely agreed position denies that mania might be induced, if not in people already vulnerable (predisposed) to BPD [8,9]. Even more important, the impossibility to diagnose Mania when it occurs during AD treatment surreptitiously reduces the rate of true BP. It is in fact well known that approximately 50% of patients have the so-called DMI (Depression-Mania-Interval) pattern, where the manic episode *naturally *follows the depressive one [10]. In spite of the reported undertreatment of depression [11], in the western world patients suffering from severe depression are likely to receive some kind of antidepressant treatment. In this case, even the Mania episode that would have occurred anyway, could not be diagnosed as Manic episode since it started during the course of AD treatment [12]. Regardless of the existence of induced Mania, it is therefore clear that a consistent part of patients are prevented from being diagnosed as BPD simply because they have depression before mania.

### False Unipolars

It is obvious that the diagnosis of BP is only possible after the occurrence of an episode of Mania/Hypomania. It is also well known that the onset of BPD occurs with Mania/hypomania in about a half of the cases. In the remaining cases, being the first episode(s) depressive (or even anxious), the initial diagnosis is unipolar depression, or other, certainly not BPD. The rate of patients that move from the diagnosis of Unipolar to that of BP is 15–30% in the literature, depending on the length of the observation period [13-15]. As the first mania may occur at any age [16], it is clear that the age of the sample conditions the rate of UP shifting to BP in epidemiological studies. For the clinician, treating a potentially BP patient poses special problems, because of the risk of switching into mania, the higher propensity to recurrences [17], the increased risk of suicide [18-20], the slower response to ADs [21]. The clinician, however, has some help in suspecting the potentially bipolar patient, even in absence of previous mania: a family history for BP, an earlier onset, more rapid recurrences, the presence of delusions, post-partum depressions, a depressive presentation with racing thoughts and/or melancholic features are all factors that have been associated with increased risk of switching to mania in the future. [14,22]. We are aware that this is a problem intrinsically connected to the distinction Unipolar-Bipolar, that none of operational diagnostic criteria can solve. It is worth reminding, however, that the category "Major Depressive Disorder" should logically be substituted by "Non Bipolar Affective Disorder", in which "non bipolar should" be read as "not yet bipolar". A transitory category, similar to that of schizophreniform disorder for schizophrenia, would probably help.

### Bipolar Spectrum

Following Klerman's classic paper on the BP Spectrum [23], several authors agree on the existence of a continuum of bipolarity, ranging with continuity from the minor, normal forms of hyperthymic temperament to the most severe cases of delusional mania. In this regard the threshold chosen by DSM IV for the diagnosis of BP would be too high, thus diagnosing as UP cases that closely resemble BPD under several clinical variables [24-26]. While this position clearly affects the epidemiological figures, it also has some relevant repercussions on the clinical practice. As said before, the patients prone to bipolarity exhibit specific risks, respond differentially to treatments, have some peculiarities that distinguish them from the true unipolars and give them some clinically diagnosable specificity. Recognizing and giving the necessary weight to the subclinical aspects of bipolarity may really improve the clinical practice. This notwithstanding, DSM IV again does not help recognizing such cases. In this regard, we agree with Carta and Angst's [1] position that the threshold currently adopted for hypomania and mixed state is too high.

As said before, we do not question the particular criteria adopted by DSM IV, that can obviously be improved, better refined or amended, but rather the possibility to properly detect the distinction between Bipolar and unipolar by rigid operational diagnostic criteria.

Quoting Kraepelin (1921)[5], *"The delimitation of the individual forms of the malady [manic depressive insanity] is in many respects wholly artificial and arbitrary. Observation not only reveals the occurrence of the gradual transitions between all the various states, but it also shows that within the shortest space of time the same morbid case may pass through most manifold transformations"*

If, as Kraepelin and many others affirm, the essential of BPD does not reside in the association of different mood states in the same individual in different periods of his life, but rather in the exceptional fluidity of affect, in the everchanging movement of emotions, in the instability and variability of mood, any still categorization is destined to fail. Freezing the emotional movement of manic depressive illness in still photographs, as it is unavoidable with diagnostic operational criteria, loses the core of the disorder and gives a false representation of it.

## Abbreviations

BPD: as Bipolar Disorder; UP: as Unipolar; AD: as Antidepressant

## Competing interests

The authors declare that they have no competing interests.

## Authors' contributions

CF had the original ideas, and revised the final manuscript, SGA, MAS, LF contributed in equal parts in writing the paper. All the authors read and approved the final manuscript

## References

[B1] Carta MG Angst J Epidemiological and clinical aspects of bipolar disorders: controversies or a common need to redefine the aims and methodological aspects of surveysj Clin Pract Epidem Ment Health 2005 1 1 4 10.1186/1745-0179-1-4 PMC1151596 15967053

[B2] Carta MG Hardoy MC Fryers T Are structured interviews truly able to detect and diagnose Bipolar II disorders in epidemiological studies: the king is still nude! Clin Pract Epidemol Ment Health 2008 4 28 19025594 10.1186/1745-0179-4-1 PMC2642514

[B3] American Psychiatric Association Diagnostic and Statistical Manual of Mental Disorders 1994 4 (DSM^IV) APA, Arlington, VA, USA

[B4] Mayer-Gross Wilhelm (Willy) Slater Eliot Roth Martin Clinical Psychiatry 1954 Cassell & Co London, UK, Ltd13199351

[B5] Kraepelin E Robertson GM, Barclay RM Manic Depressive Insanity and Paranoia 1921 Livingstone, Edinburgh, UK

[B6] Carlson GA Goodwin FK A longitudinal analysis of the manic episode Arch Gen Psychiatry 1973 28 2 221 8 4684288 10.1001/archpsyc.1973.01750320053009

[B7] Campbell JD Manic-depressive disease: Clinical and psychiatric significance 1953 Philadelphia, USA: Lippincott

[B8] Lewis JL Winokur G the induction of mania. A natural history with controls Arch Gen Psychiatry 1982 39 3 303 6 6121544 10.1001/archpsyc.1982.04290030041007

[B9] Wehr TA Goodwin FK Can antidepressants cause mania and worsen the course of affective illness? Am J Psychiatry 1987 144 11 1403 11 3314536 10.1176/ajp.144.11.1403

[B10] Goodwin JF Jamison KR Manic-depressive illness 1990 Oxford University Press, New York, NY, USA

[B11] Emilien G Septien L Brisard C Corruble E Bourin M Bipolar disorder. How far are we from a rigorous definition and effective management? Prog Neuropsychopharmacol Biol Psychiatry 2007 31 5 975 996 10.1016/j.pnpbp.2007.03.005 17459551

[B12] Goodwin GM Anderson I Arango C Bowden CL Henry C Mitchell PB Nolen WA Vieta E Wittchen HU ECNP consensus meeting. Bipolar depression. Nice, March 2007 Eur Neuropsychopharmacol 2008 18 535 549 10.1016/j.euroneuro.2008.03.003 18501566

[B13] Hirschfeld RMA Lewis L Vornik LA Perceptions and impact of BPD: how far have we really come? Results of the National Depressive and Manic-DepressiveAssociation 2000 survey of individualswithBPD J Clin Psychiatry 2003 64 161 74 12633125

[B14] Berk M Berk L Moss K Dodd S Malhi GS Diagnosing bipolar disorder: how can we do it better? Med J Aust 2006 184 459 462 16646747 10.5694/j.1326-5377.2006.tb00319.x

[B15] Angst J The bipolar spectrum British Journ of P sychiatry 2007 190 189 191 10.1192/bjp.bp.106.030957 17329735

[B16] Angst J Bipolar disorder–methodological problems and future perspectives Dialogues Clin Neurosci 2008 10 2 129 39 18689284 10.31887/DCNS.2008.10.2/jangst PMC3181871

[B17] Judd LL Akiskal HS Schettler PJ Endicott J Maser J Solomon DA Leon AC Rice JA Keller MB The long-term natural history of the weekly symptomatic status of bipolar I disorder Arch Gen Psychiatry 2002 59 530 7 10.1001/archpsyc.59.6.530 12044195

[B18] Rihmer Z Kiss K Bipolar disorders and suicidal behaviour Bipolar Disord 2002 4 Suppl 1 21 5 Review 10.1034/j.1399-5618.4.s1.3.x 12479671

[B19] Hawton K Sutton L Haw C Sinclair J Harriss L Suicide and attempted suicide in bipolar disorder: a systematic review of risk factor J Clin Psychiatry 2005 66 693 704 15960561 10.4088/jcp.v66n0604

[B20] McIntyre RS Muzina DJ Kemp DE Blank D Woldeyohannes HO Lofchy J Soczynska JK Banik S Konarski JZ Bipolar disorder and suicide: research synthesis and clinical translation Curr Psychiatry Rep 2008 10 66 72 10.1007/s11920-008-0012-7 18269897

[B21] Katzow JJ Hsu DJ Nassir Ghaemi S The bipolar spectrum: a clinical perspective Bipolar Disord 2003 5 6 436 42 10.1046/j.1399-5618.2003.00068.x 14636367

[B22] Ghaemi SN Ko JY Goodwin FK The bipolar spectrum and the antidepressant view of the world J Psychiatr Pract 2001 7 287 97 10.1097/00131746-200109000-00002 15990539

[B23] Klerman GL The spectrum of mania Comprehensive Psychiatry 1981 22 11 20 10.1016/0010-440X(81)90049-3 7460559

[B24] Akiskal HS Grinspoon L The bipolar spectrum: new concepts in classification and diagnosis Psychiatry Update 1983 2 Washington, USA: American Psychiatric Press

[B25] Akiskal HS Pinto O The evolving bipolar spectrum. Prototypes I, II, III, and IV Psychiatr Clin North Am 1999 22 517 534 10.1016/S0193-953X(05)70093-9 10550853

[B26] Cassano GB Dell'Osso L Frank E Miniati M Fagiolini A Shear K Pini S Maser J The bipolar spectrum: a clinical reality in search of diagnostic criteria and an assessment methodology J Affect Disord 1999 54 3 319 28 10.1016/S0165-0327(98)00158-X 10467978

